# P-582. Purpureocillium lilacinum Keratitis Cases Associated with an Ophthalmology Clinic — New York City, 2024

**DOI:** 10.1093/ofid/ofaf695.796

**Published:** 2026-01-11

**Authors:** Michelle E Chang, Elise Mantell, William Greendyke, Brian Schwem, Zachary Mudge, Luisa F Lopez, Dallas J Smith, Anastasia Litvintseva, Adam Rowh, Heather Moulton-Meissner, Paige Gable, Tristan D McPherson

**Affiliations:** Centers for Disease Control and Prevention, New York, NY; NYC Department of Health and Mental Hygiene, Long Island City, New York; NYC Department of Health and Mental Hygiene, Long Island City, New York; Centers for Disease Control and Prevention, New York, NY; Centers for Disease Control and Prevention, New York, NY; Centers for Disease Control and Prevention, New York, NY; Mycotic Diseases Branch, Centers for Disease Control and Prevention, Atlanta, Georgia; Centers for Disease Control and Prevention, New York, NY; Centers for Disease Control and Prevention, New York, NY; Centers for Disease Control and Prevention, New York, NY; Centers for Disease Control and Prevention, New York, NY; New York City Department of Health and Mental Hygiene, New York, NY

## Abstract

**Background:**

*Purpureocillium lilacinum*, an environmental mold, can cause sight-threatening infections after ocular surgery and demonstrates intrinsic resistance to certain antifungals. On December 18, 2024, a clinical laboratory notified the New York City Health Department of multiple patients with *P. lilacinum* keratitis after procedures at an ophthalmology clinic (Clinic A). We investigated to identify exposures associated with illness and recommend control measures.

**Methods:**

A case was defined as eye pain or vision loss in a patient after laser eye surgery at Clinic A during December 5–18, 2024, with corneal cultures demonstrating *P. lilacinum* or scrapings detecting fungal elements. We reviewed medical records, interviewed clinicians, and assessed Clinic A’s infection prevention and control (IPC) practices. We performed antifungal susceptibility testing (AFST) and whole genome sequencing (WGS) on *P. lilacinum* clinical isolates, and environmental cultures and fungal amplicon sequencing (ITS1, ITS2, and 28S rDNA) of a saline bottle, refrigerator, and surgical device for presence of *P. lilacinum*.

**Results:**

We identified 3 cases. All patients experienced vision loss; 1 required corneal transplantation. IPC assessment identified lapses in instrument reprocessing, environmental cleaning, medication safety, and exposure to nonsterile water. We observed an undated bottle of sterile saline solution used for surgical irrigation for multiple patients across multiple weeks. We gave recommendations addressing potential exposures. AFST showed resistance to amphotericin B and susceptibility to azoles. WGS showed that clinical isolates were genetically related (∼55 single nucleotide polymorphisms [SNPs] apart) but different from control isolates (∼200,000 SNPs apart). Environmental cultures were negative for *P. lilacinum* but amplicon sequencing detected *P. lilacinum* DNA in the surgical device’s suction tubing.

**Conclusion:**

*P. lilacinum* caused severe disease in all 3 patients. Whether detection of *P. lilacinum* in the surgical device supported this as the infection source or represented environmental contamination is unclear. Our investigation uncovered and addressed breakdowns in IPC practices. No further cases have been identified.

**Disclosures:**

All Authors: No reported disclosures

